# RNA Modification by m^6^A Methylation in Cardiovascular Disease

**DOI:** 10.1155/2021/8813909

**Published:** 2021-02-09

**Authors:** Jun Chen, Xiang Wei, Xin Yi, Ding-Sheng Jiang

**Affiliations:** ^1^Division of Cardiothoracic and Vascular Surgery, Tongji Hospital, Tongji Medical College, Huazhong University of Science and Technology, Wuhan, Hubei, China; ^2^Key Laboratory of Organ Transplantation, Ministry of Education, Wuhan, Hubei, China; ^3^NHC Key Laboratory of Organ Transplantation, Wuhan, Hubei, China; ^4^Key Laboratory of Organ Transplantation, Chinese Academy of Medical Sciences, Wuhan, Hubei, China; ^5^Department of Cardiology, Renmin Hospital of Wuhan University, Wuhan, Hubei, China

## Abstract

Cardiovascular disease is currently the leading cause of death worldwide, and its underlying regulatory mechanisms remain largely unknown. N^6^-Methyladenosine (m^6^A) RNA methylation is an epigenetic modification involved in the splicing, nuclear export, translational regulation, and degradation of RNA. After the initial identification of m^6^A RNA methylation in 1974, the rise of next-generation sequencing technology to detect m^6^A throughout the transcriptome led to its renewed recognition in 2012. Since that time, m^6^A methylation has been extensively studied, and its functions, mechanisms, and effectors (e.g., METTL3, FTO, METTL14, WTAP, ALKBH5, and YTHDFs) in various diseases, including cardiovascular diseases, have rapidly been investigated. In this review, we first examine and summarize the molecular and cellular functions of m^6^A methylation and its readers, writers, and erasers in the cardiovascular system. Finally, we discuss future directions for m^6^A methylation research and the potential for therapeutic targeting of m^6^A modification in cardiovascular disease.

## 1. Introduction

Cardiovascular disease remains the most common cause of death worldwide and accounts for 31.5% of all deaths [1]. Data from Europe show that more than 4 million people die from cardiovascular disease annually [1]. Similar findings have been observed in other countries, such as the United States and China [2]. The results of a global study which includes the data of 188 countries from 1990 through 2013 showed that global deaths from cardiovascular disease increased by 41% between 1990 and 2013 [3]. Scientists have performed considerable research on cardiovascular disease and have developed many useful drugs (e.g., angiotensin-converting enzyme inhibitors (ACEIs), angiotensin receptor blockers (ARBs), and statins) and treatment methods (e.g., coronary artery bypass and percutaneous coronary intervention) based on their findings [2]. These great developments have significantly reduced the mortality of patients with cardiovascular disease. However, the treatment options for cardiovascular disease are limited, and the identification and validation of new therapeutic targets for cardiovascular diseases are thus urgently needed.

To date, researchers have conducted extensive investigations on cardiovascular disease at the genetic and epigenetic levels, and these studies have greatly deepened our understanding of cardiovascular disease [4–11]. Most conventional therapies available for patients with cardiovascular disease involve protein-targeting drugs. Before mRNA is translated into protein, it is regulated via various mechanisms at the transcriptional and posttranscriptional levels. N^6^-Methyladenosine (m^6^A) RNA methylation is methylation that occurs in the N^6^-position of adenosine, which is an RNA epigenetic modification that regulates RNA at the posttranscriptional level [12–14]. m^6^A RNA methylation regulates not only the splicing and translation but also the stability of RNA [15]. Recently, the critical role of m^6^A in cardiovascular diseases has been highlighted [16], and in this review, we summarize the research advances in our understanding of m^6^A-dependent biological functions and molecular mechanisms in cardiovascular disease.

## 2. Discovery and Regulation of m^6^A Methylation

**Figure 1:**
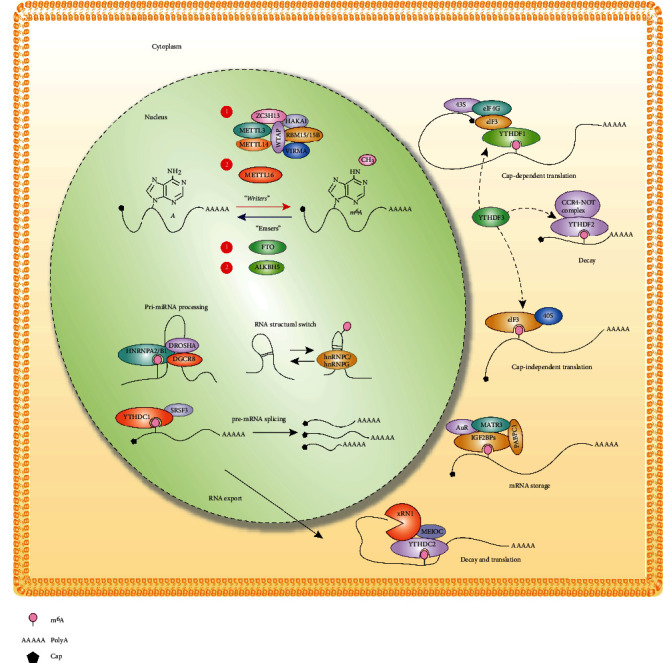
Schematic diagram illustrating the effectors of m^6^A RNA methylation and their impacts on RNA regulation. The writer, eraser, and reader proteins that regulate gene expression via m^6^A RNA methylation are shown. m^6^A RNA methylation is involved in pri-miRNA processing, RNA structural switching, pre-mRNA splicing, RNA export, and mRNA storage, translation, and decay.

m^6^A RNA methylation was first identified in poly(A) RNA fractions by two independent groups in 1974 [14, 17], but research on m^6^A did not attract substantial attention because methods for detecting m^6^A sites in RNAs were lacking. m^6^A research was revived in 2012 with the emergence of next-generation sequencing technology to detect m^6^A throughout the transcriptome [18, 19]. The current research findings indicate that RNA m^6^A is found widely across species [20–22]. Indeed, more than 10,000 m^6^A sites have been identified in over 25% of human transcripts; these sites are commonly located in 3′ untranslated regions (3′ UTRs), adjacent to stop codons, and, in long exons, while varied levels of m^6^A are also found in regions surrounding start codon and 5′ UTRs [18, 19, 23].

m^6^A is dynamically regulated by its modification “effectors,” including “writers,” “erasers,” and “readers,” which install and remove methylation or recognize chemical marks, respectively [24, 25]. Methyltransferase-like 3 (METTL3), METTL14, Wilms' tumor 1-associated protein (WTAP), and KIAA1429 form a multicomponent methyltransferase complex, of which METTL3 is a subunit with methyltransferase activity that functions as a writer to install m^6^A on RNA molecules [26–30]. After the initial identification of the m^6^A demethylase fat mass and obesity-associated protein (FTO) in 2011 [31], AlkB homolog 5 (ALKBH5) was also shown to have demethylase activity [32]. ALKBH5 and FTO exhibit m^6^A eraser activity under different pathophysiological conditions and potentially regulate different subsets of target RNAs due to their distinct subcellular and tissue distributions [32, 33]. After RNA is methylated by m^6^A writers, readers, including YT521-B homology (YTH) domain family proteins (YTHDF1, YTHDF2, YTHDF3, and YTHDC1) and heterogeneous nuclear ribonucleoprotein family proteins (HNRNPA2B1 and HNRNPC), bind to and recognize methylated RNAs and determine their fate [34–39]. Studies have shown that m^6^A is a highly pervasive modification that affects RNA splicing, export, translation, and stability to regulate cell differentiation, embryonic development, stress responses, and diseases [18, 19, 25, 34, 35, 40–42] (Figure 1). Several published reviews have summarized the regulatory mechanisms of m^6^A, as well as its role and mechanism in tumors [12, 15, 22, 24, 43]; therefore, we will not discuss these topics in detail. Interested readers can read the relevant reviews. In the present review, we focus on these recent advances in the understanding of how m^6^A methylation and its writers, erasers, and readers affect cardiovascular disease, and we also propose some new research ideas and future directions based on the available knowledge in this field.

## 3. m^6^A Methylation in Heart-Related Diseases

### 3.1. Genetic Polymorphisms in m^6^A Regulators in Heart Diseases

#### 3.1.1. Polymorphisms in the m^6^A Eraser Fat Mass and Obesity-Associated Protein (FTO)

Gene mutation is a common etiology of cardiovascular diseases (e.g., dilated cardiomyopathy, hypertrophic cardiomyopathy, and congenital heart disease). Specific variants in *FTO* have been reported to predispose individuals to type 2 diabetes mellitus (T2DM) and obesity, which are common risk factors for cardiovascular diseases [44–47]. Among these variants, the *FTO* rs9939609 (T>A) polymorphism is the most thoroughly investigated. In two Swedish population-based case-control studies, individuals with the *FTO* rs9939609 (T>A) variant exhibited an increased risk of coronary heart disease (CHD) after adjustment for sex, age, and body mass index (BMI). Moreover, this association was not offset by increased physical activity [48]. Similarly, in the Oulu Project Elucidating Risk of Atherosclerosis (OPERA) study, both Cox regression analysis and logistic regression analysis demonstrated that the incidence of CHD (odds ratio (OR) = 1.905) and cardiovascular disease events or death (OR = 1.849) were strikingly higher in individuals with the *FTO* rs9939609 minor allele variant [49]. In contrast, the minor allele (A) variant of *FTO* rs9939609 showed no obvious correlation with CHD in a case-control study conducted in the Iranian population [50]. This apparent discrepancy may be explained by differences in race, lifestyle, or environment.

Several studies have been conducted to further investigate how the *FTO* rs9939609 variant affects cardiovascular disease through interaction with factors such as race and lifestyle. For example, Gustavsson et al. demonstrated no evidence of an interaction between macronutrient intake and *FTO* genotype on BMI or the risk of CHD [51]. In contrast, in a large-scale meta-analysis of 40 studies, the *FTO* rs9939609 variant was associated with increased BMI in Asians, Caucasians, and all participants combined but not in African-Americans [52].

In addition, the *FTO* rs1421085 (C>T) variant was demonstrated to be associated with macronutrient intake in a genome-wide meta-analysis [53]. Interestingly, Hubacek et al. revealed that the *FTO* rs17817449 variant was associated with transplant rejection in heart transplant patients [54]. These findings suggest that *FTO* polymorphisms are involved in or even act as independent risk factors for cardiovascular disease. Furthermore, it is very important to reveal the exact role of *FTO* polymorphisms in cardiovascular disease via *in vivo* knock-in of mutant *FTO* in order to provide biological support to the genomic association studies.

#### 3.1.2. Polymorphisms/Mutations in the m^6^A Readers YT521-B Homology (YTH) Domain Family Protein 3 (YTHDF3) and Heterogeneous Nuclear Riboprotein A1 (Hnrnpa1)

A genome-wide association study demonstrated that the *YTHDF3* rs4739066 variant was weakly associated with myocardial infarction in the Saudi Arabian population [55]. Yu et al. identified a novel recessive frameshift mutation in *HNRNPA1* (*HNRNPA1*^ct^) in subjects with congenital heart disease and established an *Hnrnpa1*^ct/ct^ homozygous mutant mouse model that exhibited complete penetrance of congenital heart defects [56]. Similarly, mice with global *Hnrnpa1* knockout (*Hnrnpa1*^−/−^) showed embryonic lethality due to defects in muscle development [57]. These results indicate that Hnrnpa1 is indispensable for embryonic heart development in mice and humans. Because multiple RNA m^6^A modifiers are associated with cardiovascular disease, the exact role of RNA m^6^A and its modification enzymes in cardiovascular diseases has attracted considerable attention.

### 3.2. m^6^A Methylation Landscape in Heart Diseases

**Figure 2:**
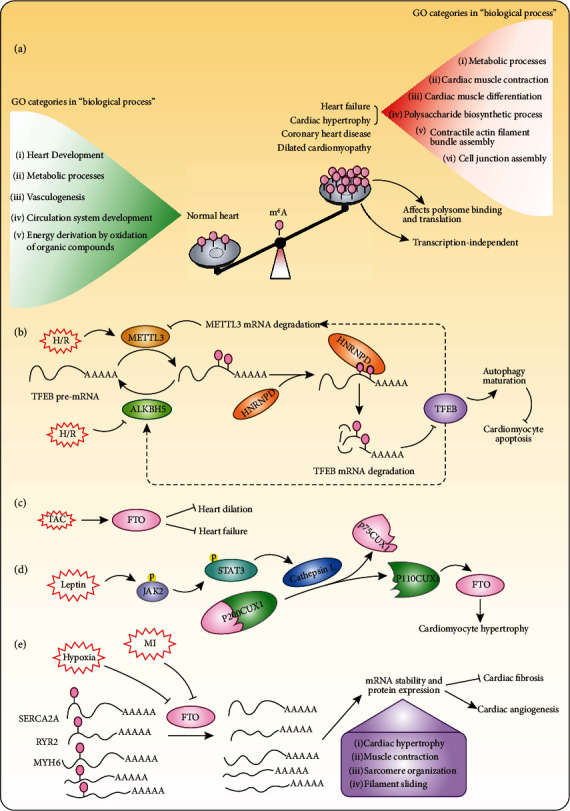
Mechanisms of m^6^A RNA methylation in cardiac disease. (a) Biological processes regulated by m^6^A RNA methylation during the development of several kinds of heart diseases. (b) METTL3 and ALKBH5 inversely regulate m^6^A modification of TFEB mRNA to affect its stability, which regulates cardiomyocyte autophagy and apoptosis under hypoxia/reoxygenation (H/R). (c) FTO inhibits pressure overload-induced heart dilation and failure. TAC: transverse aortic constriction. (d) Leptin induces FTO upregulation in cardiomyocytes via JAK2/STAT3-dependent CUX1 upregulation, and FTO upregulation results in the hypertrophic response. (e) Hypoxia and myocardial infarction (MI) suppress FTO expression which selectively demethylates cardiac contractile transcripts (e.g., SERCA2A, RYR2, and MYH6) to prevent their degradation. FTO overexpression decreases fibrosis and enhances angiogenesis in mouse models of MI.

*Mettl3* global knockout (*Mettl3*-KO) in mice has been shown to lead to early embryonic lethality at E8.5, and the KO embryos were deformed and lost the typical postimplantation epiblast egg cylinder shape at E5.5 to E7.5, indicating an essential role for m^6^A methylation during embryonic development [58]. To determine the function of m^6^A RNA methylation in cardiovascular disease, m^6^A methylation levels were detected in samples from patients (e.g., patients with dilated cardiomyopathy or CHD) or mice subjected to pathological stress (e.g., pressure overload and ischemia/reperfusion (I/R) injury) via m^6^A-specific methylated RNA immunoprecipitation followed by next-generation sequencing (MeRIP-Seq) [59–62]. The results of multiple independent research teams have demonstrated that m^6^A methylation levels are markedly increased in several cardiovascular diseases, such as heart failure, cardiac hypertrophy, and myocardial infarction [59–62] (Figure 2(a)), indicating that m^6^A methylation is associated with cardiovascular disease development.

To explore the landscape of m^6^A RNA methylation during the development of heart failure, Berulava et al. performed MeRIP-Seq both in failing human hearts and in mice subjected to transverse aortic constriction (TAC) [63]. Their results revealed that 24% of all detected transcripts (3208 peaks linked to 2164 transcripts) carried m^6^A marks, which were primarily located in the 5′ UTR or 3′ UTR and the coding sequence (CDS). Gene ontology (GO) analysis indicated that overlapping m^6^A transcripts in humans and mice were linked to cardiac muscle contraction, cardiac muscle differentiation, and metabolic processes. Interestingly, in both mice and humans, the m^6^A-methylated transcripts differed substantially from those transcripts affected at the transcriptional level of expression, suggesting that m^6^A RNA methylation did not control gene transcriptional regulation during heart failure. Given that m^6^A RNA methylation can regulate mRNA translation by affecting ribosome occupancy, their further study demonstrated that altered m^6^A methylation of mRNA regulates protein levels by affecting polysome binding of the corresponding cardiac transcripts in a transcription-independent manner and that these RNAs were linked mainly to metabolic and regulatory pathways [63]. However, the effects of m^6^A methylation on protein expression were confirmed only on an individual basis via western blot analysis. High-throughput proteomics would be a better approach to illustrate the relationship between m^6^A methylation and transcription-independent protein expression.

### 3.3. Methyltransferase-Like 3 (METTL3) in Heart Diseases

The observations that the level of m^6^A methylation is significantly increased in several types of heart disease and that m^6^A methylation is enriched mainly on specific target genes related to heart disease suggest that m^6^A modifiers may play an important regulatory role in heart disease. Dorn et al. demonstrated that cardiac-specific *Mettl3*-overexpressing (*Mettl3*-TG) mice exhibited a significant increase in m^6^A RNA levels in cardiomyocytes and that dose-dependent cardiac hypertrophic growth was detected as early as 3 months of age [59]. Structural but not histopathologic changes were observed in the hearts of *Mettl3*-TG mice at the age of 8 months. Moreover, when challenged with pressure overload, the hypertrophic response was not exacerbated by *Mettl3* overexpression at the organ level or in the cardiomyocyte cross-sectional area. These results indicated that METTL3 upregulation induced compensated hypertrophic cardiac remodeling without cardiac dysfunction either at baseline or under pressure overload [59]. Consistent with the results in *Mettl3*-TG mice, no histopathologic or hypertrophic changes were observed in *Mettl3* conditional knockout (*Mettl3*-cKO) mice at the age of 3 months. However, *Mettl3*-cKO mice begin to show cardiac abnormalities consistent with progression toward heart failure at 8 months of age [59]. In contrast, Kmietczyk et al. reported a different result: *Mettl3* overexpression-mediated by adeno-associated virus 9 (AAV9) in mice inhibited pathological hypertrophic cellular growth and fibrosis induced by pressure overload [60]. Their results also demonstrated that m^6^A affects translational efficiency but not RNA transcription during pathological growth, but the exact mechanisms by which METTL3 regulates cellular growth and the specific targets responsible for the phenotypic consequences remain unknown [60]. Recently, Gao et al. demonstrated that a cardiac-hypertrophy-associated piRNA (CHAPIR) directly interacts with METTL3 to upregulate PARP10 expression via blocking the m^6^A methylation of Parp10 mRNA transcripts to facilitate pathological hypertrophy and cardiac remodelling [64]. As Kmietczyk et al. explained in their article, the possible reasons for these differences include the different model animals used (FVB background mice in the Dorn study and C57BL6/N mice in the Kmietczyk study) and different approaches used to overexpress METTL3 (a cardiac-specific transgene approach in the Dorn study and an AAV9-based approach in the Kmietczyk study) [60].

In addition to its role in cardiac hypertrophy, METTL3 has also been shown to play a critical role in myocardial infarction. METTL3 and myocardial m^6^A modification levels were significantly elevated in heart tissues from infarct patients compared with the levels in control tissues, and similar results were observed in H9C2 and neonatal mouse ventricular cardiomyocytes subjected to hypoxia/reoxygenation (H/R) as well as in mouse myocardial tissues subjected to I/R [61]. Further *in vitro* studies demonstrated that knockdown of METTL3 facilitates autophagic flux but inhibits apoptosis in cardiomyocytes subjected to H/R, while overexpression of METTL3 or silencing ALKBH5 has the opposite effect. Mechanistically, METTL3 methylates transcription factor EB (TFEB) at two m^6^A residues in the 3′ UTR, which subsequently decreases the expression levels of TFEB. Interestingly, TFEB directly binds to the *Alkbh5* promoter to activate its transcription and inhibits METTL3 expression by downregulating mRNA stability in a negative feedback loop [61] (Figure 2(b) and Table 1). However, although the authors reported that *Mettl3* knockout mice had no abnormalities in cardiac structure or function under normal conditions, they did not evaluate cardiac changes after myocardial infarction or I/R injury *in vivo* [61].

The results of the above studies suggest that different stimuli and even different *Mettl3* overexpression methods modulate the function of METTL3. The regulation of different target genes under different stimuli, the genetic background of mice, the specificity of overexpression strategies, and differences in *in vivo* and *in vitro* experiments may all be the reasons for the controversial results of METTL3. Therefore, conducting more in-depth research to determine the function and mechanisms of METTL3 in the cardiovascular system by using mice with cell-specific Cre- (such as *Nkx2-5*-Cre, *cTNT*-cre, and *Mef2c*-AHF-cre) mediated *Mettl3* knockout under the same stimulation conditions or to identify its effects on other cardiovascular diseases (e.g., diabetic cardiomyopathy, aortic aneurysm/dissection, atherosclerosis, and pulmonary arterial hypertension) is warranted.

### 3.4. Fat Mass and Obesity-Associated Protein (FTO) in Heart Diseases

Unlike global *Mettl3* knockout, *Fto* knockout mice (*Fto*^−/−^) is not embryonically lethal; however, compared to their wild-type counterparts, *Fto*^−/−^ mice are characterized by growth retardation, increased heart rates, altered ventricular repolarization, enhanced vulnerability to stress-induced tachyarrhythmias, and cardiac hypertrophy [65]. This pattern indicates that FTO is involved in cardiac pathophysiology. Indeed, Berulava et al. investigated the role of the m^6^A demethylase FTO in heart failure in a cardiac-specific *Fto* knockout (*Fto*-cKO) mouse model and observed a more severe reduction in the ejection fraction and a higher degree of dilatation in *Fto*-cKO mice subjected to TAC surgery [63] (Figure 2(c) and Table 1). However, an earlier study arrived at the opposite conclusion. Gan et al. demonstrated that FTO was upregulated via a JAK2/STAT3-CUX1-dependent pathway under leptin treatment and that siRNA-mediated knockdown of *Fto* abrogated leptin-induced cardiomyocyte hypertrophy [66] (Figure 2(d) and Table 1). Similarly, hypertrophy of neonatal rat cardiomyocytes (NRCMs) was found to be blunted by *Fto* knockdown in response to *α*-adrenergic stimulation with phenylephrine (PE) [60]. These findings suggest that the effects of FTO on cardiac hypertrophy may be stimulation-dependent, and the exact molecular mechanism needs to be further clarified. Moreover, whether the demethylase activity of FTO is necessary for this function needs further investigation.

Fortunately, a recently published study may provide some answers [62]. First, both the mRNA and protein levels of FTO were found to be significantly decreased in failing left ventricular (LV) explants from human, pig, and mouse compared to the respective levels in nonfailing or sham explants; these decreases were accompanied by an increase in m^6^A methylation levels in infarct and peri-infarct regions but not in noninfarcted (remote) left ventricular tissue. Second, AAV9-mediated overexpression of *Fto* improves cardiac function post myocardial infarction and reduces fibrosis in mice, demonstrating the therapeutic potential of FTO. Third, gain- and loss-of-function studies with MeRIP-Seq showed that FTO demethylates a subset of transcripts largely associated with muscle contraction, sarcomere organization, filament sliding, and cardiac hypertrophy in primary cardiomyocytes. Specifically, FTO selectively demethylates cardiac contractile transcripts such as SERCA2A, MYH6/7, RYR2, and many others to increase their mRNA stability and protein expression (Figure 2(e) and Table 1). Thus, these results suggest that FTO plays an important functional role in cardiac homeostasis and myocardial repair. Introducing a demethylase-inactivating mutation in FTO would help to further clarify whether the function of FTO in cardiac homeostasis is dependent on its demethylase activity.

## 4. m^6^A Methylation in Vascular Diseases

m^6^A methylation not only regulates cardiac disease but also contributes to arterial disease. In a recently published study, He et al. demonstrated that the m^6^A level was significantly higher in the aortic tissue of patients with abdominal aortic aneurysm (AAA) than in healthy aortic tissue and that the m^6^A level was positively associated with the risk of AAA rupture [67]. Furthermore, patients with higher YTHDF3 expression had a greater risk of AAA rupture, METTL14 expression was associated with inflammatory infiltrates and neovascularization, and FTO expression was strongly correlated with YTHDF3 expression, aneurysmal SMCs, and macrophage infiltration [67]. These results indicate that m^6^A methylation and its regulators are essential for the inflammatory response in the vascular system; indeed, many vascular diseases (e.g., atherosclerosis) are accompanied by inflammation.

### 4.1. Genetic Polymorphisms in m^6^A Regulators in Vascular Diseases

Although the prevalence of *FTO* variants (rs8050136 and rs9939609) does not differ significantly among patients with large artery atherosclerotic stroke and control subjects [68], *FTO* variants have been reported to affect some risk factors for plaque formation. For example, in postmenopausal women, *FTO* rs9939609 was found to be more prevalent in individuals with higher triglyceride, sCD40L, and visfatin levels and was also associated with higher homocysteine levels, BMI, and total cholesterol levels [69, 70]. Moreover, overexpression of *Fto* was found to inhibit cholesterol ester accumulation in macrophages treated with oxidized LDL, while *Fto* deficiency exerted the opposite effects. Furthermore, AAV9-mediated *Fto* overexpression markedly reduced the contents of plasma total cholesterol and LDL cholesterol and suppressed the formation of atherosclerotic plaques in *ApoE*^−/−^ mice [71]. In obese mice and humans, FTO expression levels are elevated in vascular tissue [72]. Selective deletion of endothelial *Fto* in mice did not influence the development of obesity and dyslipidemia but did reduce the development of high-fat diet-induced glucose intolerance, insulin resistance, and hypertension [72]. In addition, variants of *FTO* (rs1121980, rs1421085, and rs17817449) have been reported to be significantly associated with diabetic nephropathy after stratification of patients according to the presence/absence of vascular complications [73]. The aforementioned results suggest that FTO is associated with multiple vascular conditions; however, the precise function and molecular mechanisms of FTO in various vascular diseases (e.g., aortic dissection/aneurysm, neointimal formation, and pulmonary arterial hypertension) are unclear.

### 4.2. Methyltransferase-Like 3 (METTL3) in Vascular Diseases

**Figure 3:**
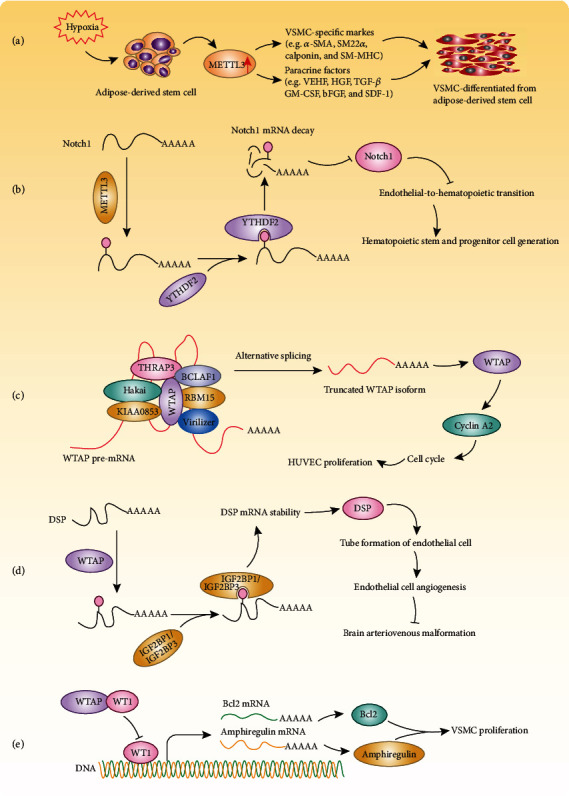
Mechanisms of m^6^A RNA methylation in vascular disease. (a) METTL3 facilitates adipose-derived stem cell differentiation into vascular smooth muscle cells (VSMCs) by regulating VSMC-specific markers and paracrine factors. (b) METTL3 methylates Notch1 mRNA which is read by YTHDF2 leading to degradation of Notch1 mRNA and promoting hematopoietic stem and progenitor cell generation through endothelial-to-hematopoietic transition. (c) WTAP forms a protein complex with Hakai, Virilizer homolog, KIAA0853, RBM15, BCLAF1, and THRAP3 to regulate cyclin A2 mRNA stability in order to modulate human umbilical vein endothelial cell (HUVEC) proliferation. (d) WTAP facilitates DSP mRNA m^6^A methylation, preventing its degradation, to promote endothelial cell angiogenesis and tube formation, thus inhibiting brain arteriovenous malformation. (e) WTAP interacts with WT1 to inhibit BCL2 and amphiregulin expression, which suppresses smooth muscle cell (VSMC) proliferation but facilitates VSMC apoptosis.

m^6^A methyltransferases have also been reported to play vital roles in vascular biology. For example, Lin et al. found that METTL3 is essential for the differentiation of adipose-derived stem cells into vascular smooth muscle cells (VSMCs) [74]. METTL3 was found to be upregulated in VSMCs differentiated from adipose-derived stem cells. Moreover, silencing *METTL3* reduced the expression levels of VSMC-specific markers (SM22*α*, calponin, *α*-SMA, and SM-MHC) and paracrine factors (HGF, VEGF, TGF-*β*, bFGF, GM-CSF, and SDF-1), indicating that METTL3 facilitates the differentiation of hypoxia stress-induced adipose-derived stem cells into VMSCs [74] (Figure 3(a) and Table 1). METTL3 not only plays a role in the differentiation of adipose-derived stem cells into VSMCs but also is critical for hematopoietic stem and progenitor cell (HSPC) development during definitive hematopoiesis in mice. In mice with specific knockout of *Mettl3* in endothelial cells of the aorta-gonad-mesonephros (AGM) region (*Mettl3*^fl/fl^/Vec-Cre), the proportions of HSPCs (CD34^+^c-Kit^+^), pre-HSCs (CD144^+^CD45^+^c-Kit^+^), and hemogenic endothelial cells (CD31^+^c-Kit^+^) were apparently reduced in the AGM of E10.5 *Mettl3*^fl/fl^/Vec-Cre mice, and the colony-forming ability and short-term reconstitution capability of HSPCs were diminished. Mechanistically, METTL3-mediated m^6^A methylation on Notch1 mRNA, which is recognized by YTHDF2, results in decay of Notch1 mRNA, inhibiting Notch activity in hemogenic endothelial cells and thereby promoting HSPC generation through endothelial-to-hematopoietic transition [75] (Figure 3(b) and Table 1). Interestingly, *Mettl3* deficiency not only reduced the expression level of VEGFA but also decreased the mRNA level of its splice variants, VEGFA-164 and VEGFA-188, in bone marrow mesenchymal stem cells to regulate osteogenic differentiation [76]. Given that the VEGFA signaling pathway is very important for angiogenesis, vascular permeability, and spermatogonial stem cell maintenance, METTL3 is likely to have an indispensable effect on these biological processes [77, 78]. In addition, METTL14 expression levels were found to be increased in calcified arteries and primary human artery smooth muscle cells (HASMCs) induced by indoxyl sulfate. Knockdown of *METTL14* decreases the RNA m^6^A level and attenuates indoxyl sulfate-induced HASMC calcification [79].

### 4.3. Wilms Tumor 1-Associated Protein (WTAP) in Vascular Diseases

WTAP is an important regulatory subunit of the RNA methyltransferase complex and is indispensable for embryonic development, because homozygous *Wtap*^−/−^ knockout is embryonic lethal in mice [80]. *WTAP* knockdown was found to induce G_2_ accumulation of human umbilical vein endothelial cells (HUVECs), resulting in markedly reduced expression of cyclin A2 [80] (Figure 3(c) and Table 1). Furthermore, in HUVECs, WTAP forms a protein complex with Hakai, Virilizer homolog, KIAA0853, RBM15, BCLAF1, and THRAP3 to function as an RNA processing machine [81]. Alternative splicing of *WTAP* pre-mRNA is regulated by this WTAP complex to promote the production of a truncated WTAP isoform, thus affecting WTAP protein expression [81]. The WTAP complex is critical for cell proliferation, because depletion of individual components of the complex resulted in G_2_/M delay [81].

Recently, Wang et al. found that WTAP was downregulated in brain arteriovenous malformation lesions, which are congenital vascular abnormalities characterized by direct connections among arteries and veins without an intervening capillary bed [82]. In addition, knockdown of *Wtap* inhibits endothelial cell angiogenesis *in vitro* by significantly reducing the mRNA level of desmoplakin (DSP), which affects the tube formation of endothelial cells. MeRIP-seq and m^6^A-IP-qPCR results demonstrated that m^6^A enrichment on DSP was largely abolished in endothelial cells with *Wtap* deficiency and that the half-life of DSP mRNA was dramatically shortened. These results indicate that WTAP can affect the stability of DSP mRNA by promoting its m^6^A methylation, and m^6^A-methylated DSP mRNAs are recognized by insulin like growth factor 2 mRNA binding protein 1 (IGF2BP1) and IGF2BP3 to prevent their degradation [82] (Figure 3(d) and Table 1). However, a different pattern was observed in SMCs [83]. First, in contrast to the increase in WTAP expression in endothelial cells during proliferation, the WTAP expression level was higher in nonproliferating SMCs, and in the intima of injured arteries, WTAP was upregulated in the late stages of repair. Second, knockdown of endogenous *Wtap* facilitated SMC proliferation, promoting DNA synthesis and G1/S transition but suppressing apoptosis, while overexpression of *Wtap* produced the opposite effect, and amphiregulin and Bcl-2 were found to mediate the antiproliferative effect of WTAP [83, 84] (Figure 3(e) and Table 1).

In addition, the IGF-1/PI3K/Akt signaling axis directed WTAP degradation via the nuclear 26S proteasome to reduce the abundance of WTAP, which led to a switch in the Survivin splice variant from proapoptotic Survivin-2B to antiapoptotic Survivin in VSMCs [85]. Considering the different functions of WTAP in SMCs and endothelial cells, WTAP, which can not only inhibit excessive SMC proliferation but also promote endothelialization, may be an ideal target for the treatment of neointima formation to prevent restenosis. Therefore, *in vivo* transgenic mouse models with inducible and cell-specific WTAP expression are urgently needed to evaluate this possibility.

### 4.4. m^6^A Readers in Vascular Diseases

In addition to m^6^A writers and erasers, m^6^A readers also have functions in vascular biology. For example, both the mRNA and protein expressions of hnRNPA2/B1 were found to be significantly upregulated in embryonic stem cells after 3 to 7 days of incubation in SMC differentiation medium [86]. Moreover, hnRNPA2/B1 can transcriptionally regulate *α*-SMA and SM22*α* expression through direct binding to their promoters. An hnRNPA2/B1 morpholino specifically designed for *in vivo* knockdown suppressed the differentiation of neural crest cells into SMCs in chick embryos, which led to maldevelopment of branchial arch arteries and increased embryo lethality at a later developmental stage [86]. These results indicated that hnRNPA2/B1 is a potential target for vascular regenerative medicine (Table 1). In addition, YTHDF2 plays a critical role in vascular reconstruction in hepatocellular carcinoma (HCC) [87]. Silencing of *YTHDF2* in human HCC cells or ablation of *Ythdf2* in mouse hepatocytes was found to aggravate inflammation, vascular reconstruction, and metastatic progression via decay of m^6^A-containing interleukin 11 and serpin family E member 2 (SERPINE2) mRNAs (Table 1).

Although some studies have addressed m^6^A methylation in the context of vascular biology, the field is still in its infancy, and many scientific issues need further study. For example, how do m^6^A levels change under different vascular conditions? Is methyltransferase or demethylase activity essential for the functions of these enzymes in vascular biology or diseases? More importantly, the functions and mechanisms of m^6^A modulators in various vascular diseases (e.g., atherosclerosis, neointima formation, aortic aneurysm/dissection, and pulmonary arterial hypertension) have not been elucidated.

## 5. Conclusion and Perspectives

Published studies suggest that m^6^A regulators, especially METTL3 and FTO, play important roles in a variety of cardiovascular diseases, but many scientific issues have not elucidated. First, determining whether m^6^A methylation profiles differ according to the etiology of cardiovascular diseases would be very interesting. Future studies should focus on the mechanisms that determine which RNAs become hyper- or hypomethylated and the effects of these changes on RNA fate during biological processes of cardiovascular diseases. Second, whether methyltransferase or demethylase activity is indispensable for the impact of these enzymes on cardiovascular diseases must be verified. Third, some variants of m^6^A writers, erasers, and readers in humans have been reported to be closely related to cardiovascular disorders, but the precise function of these polymorphisms in cardiovascular diseases remains unknown. Knock-in animal models would be useful to address this issue. Fourth, the roles of m^6^A methylation and its regulatory writers, erasers, and readers in various cardiovascular diseases, such as diabetic cardiomyopathy and pulmonary arterial hypertension, are unclear.

In addition, several high-throughput methods (e.g., MeRIP, m^6^A-seq, and PA-m^6^A-seq) are available to detect m^6^A, but detecting m^6^A sites at single-nucleotide resolution remains challenging. The m^6^A individual-nucleotide resolution crosslinking and immunoprecipitation (miCLIP) approach can detect m^6^A sites at single-nucleotide resolution, but cannot differentiate RNA modifications (e.g., m^1^A and m^6^A) that occur simultaneously in the same RNA molecule [88]. In addition, a major limitation of these approaches is the promiscuity of anti-m^6^A antibodies, and devising appropriate methods to eliminate false positives is challenging. To gain a detailed understanding of the mechanisms by which m^6^A dictates the fate of mRNAs and their roles in cardiovascular disease, we must identify the specific mRNA sites that are m^6^A modified under a given pathophysiological condition. Furthermore, we must develop new and better tools or methods to more precisely manipulate the m^6^A methylation of specific transcripts at specific sites in order to better understand the mechanisms by which m^6^A influences the fate of RNAs, which will help guide the development of therapies targeting m^6^A writers, erasers, or readers to safely and effectively manipulate m^6^A methylation for cardiovascular disease therapy.

## Figures and Tables

**Table 1:** Roles and mechanisms of RNA m^6^A effectors in cardiovascular disease.

RNA m^6^A effectors	Methylated RNA targets or RNA fate regulation	Functions in cardiovascular disease	References
Methyltransferase-like 3 (METTL3)	Not known	*Mettl3*-Tg mice show compensated cardiac hypertrophic remodeling without cardiac dysfunction at both baseline and under pressure overload. However, *Mettl3*-cKO mice show eccentric hypertrophy, increased left ventricular chamber dimensions and ventricular dilation, and reduced cardiac function.	[59]
METTL3	Regulates mRNA translational efficiency	Adeno-associated virus 9- (AAV9-) mediated Mettl3 overexpression in mice inhibits pathological hypertrophic cellular growth and fibrosis induced by pressure overload.	[60]
METTL3/AlkB homolog 5 (ALKBH5)/heterogeneous nuclear ribonucleoprotein D (HNRNPD)	Installs m^6^A in TFEB at two residues in the 3′ UTR, leading to degradation of its mRNA	METTL3 inhibits autophagic flux but facilitates apoptosis in cardiomyocytes subjected to hypoxia/reoxygenation. ALKBH5 exerts the opposite effects.	[61]
Fat mass and obesity-associated protein (FTO)	Not known	*Fto*-cKO mice show a more severe reduction in the ejection fraction and a higher degree of dilatation after pressure overload.	[63]
FTO	Not known	*Fto* knockdown abrogates leptin-induced cardiomyocyte hypertrophy and inhibits phenylephrine-induced hypertrophy of neonatal rat cardiomyocytes.	[60, 66]
FTO	Demethylates SERCA2A, MYH6/7, and RYR2 mRNA, increasing the stability and protein expression of these mRNAs	AAV9-mediated overexpression of *Fto* improves cardiac function post myocardial infarction and reduced fibrosis in mice.	[62]
METTL3	Not known	METTL3 facilitates the differentiation of hypoxia stress-induced adipose-derived stem cells into vascular smooth muscle cells.	[74]
METTL3/YT521-B homology (YTH) domain family protein 2 (YTHDF2)	Methylates Notch1 mRNA, resulting in its decay	METTL3 promotes hematopoietic stem and progenitor cell generation through endothelial-to-hematopoietic transition.	[75]
Wilms tumor 1-associated protein (WTAP)	Regulates cyclin A2 mRNA stability	*Wtap* knockdown induces G_2_ accumulation of human umbilical vein endothelial cells.	[80]
WTAP/insulin like growth factor 2 mRNA binding protein 1 (IGF2BP1)/IGF2BP3	Methylates desmoplakin (DSP) mRNA, preventing its degradation	Knockdown of *Wtap* inhibits endothelial cell angiogenesis and tube formation.	[82]
WTAP	Not known	WTAP inhibits the proliferation but facilitates the apoptosis of smooth muscle cells.	[83]
hnRNPA2/B1	Not known	hnRNPA2/B1 knockdown suppresses the differentiation of neural crest cells into smooth muscle cells, leading to maldevelopment of branchial arch arteries in chick embryos.	[86]
YTHDF2	Inhibits the decay of m^6^A-containing interleukin 11 and serpin family E member 2 (SERPINE2) mRNAs	Silencing of *YTHDF2* in hepatocellular carcinoma cells or ablation of *Ythdf2* in mouse hepatocytes aggravates inflammation, vascular reconstruction, and metastatic progression.	[87]
